# Induction of neutralizing antibodies specific for the envelope proteins of the koala retrovirus by immunization with recombinant proteins or with DNA

**DOI:** 10.1186/s12985-015-0296-2

**Published:** 2015-04-30

**Authors:** Uwe Fiebig, Britta Dieckhoff, Christian Wurzbacher, Annekathrin Möller, Reinhard Kurth, Joachim Denner

**Affiliations:** Robert Koch-Institute, Nordufer 20, D-13353 Berlin, Germany

**Keywords:** Retroviruses, Koala, Transmembrane envelope proteins, Neutralizing antibodies

## Abstract

**Background:**

The koala retrovirus (KoRV) is the result of a transspecies transmission of a gammaretrovirus with fatal consequences for the new host. Like many retroviruses, KoRV induces lymphoma, leukemia and an immunodeficiency that is associated with opportunistic infections in the virus-infected animals. We recently reported the induction of neutralizing antibodies by immunization with the recombinant ectodomain of the transmembrane envelope protein p15E of KoRV. Since the neutralization titers of the p15E-specific sera were only moderate, we investigated the use of the surface envelope protein gp70 to induce neutralizing antibodies.

**Findings:**

We immunized rats and goats with the recombinant gp70 protein of the KoRV, an unglycosylated protein of 52kD (rgp70/p52) or with the corresponding DNA. In parallel we immunized with recombinant rp15E or with a combination of rp15E and rgp70/p52. In all cases binding and neutralizing antibodies were induced. The gp70-specific sera had titers of neutralizing antibodies that were 15-fold higher than the p15E-specific sera. Combining rp15E and rgp70/p52 did not significantly increase neutralizing titers compared to rgp70/p52 alone. High titers of neutralizing antibodies specific for gp70 were also induced by immunization with DNA. Since KoRV and PERV are closely related, we investigated cross-neutralization of the antisera. The antisera against p15E and gp70 of PERV and KoRV inhibited infection by both viruses.

**Conclusion:**

The envelope proteins of the KoRV may therefore form the basis of an effective preventive vaccine to protect uninfected koalas from infection and possibly an immunotherapeutic treatment for those already infected.

**Electronic supplementary material:**

The online version of this article (doi:10.1186/s12985-015-0296-2) contains supplementary material, which is available to authorized users.

## Background

Horizontal transmission of retroviruses between different species is common. In many cases, e.g., HIV-1 and HIV-2 transmitted from non-human primates [1], these transspecies transmissions cause fatal immunodeficiency disease. The koala retrovirus (KoRV) is a more recently described example of a transspecies transmission [2]. The virus was transmitted to koalas, possibly from rodents [3-6] or bats [7,8], it induces leukemia in infected animals [9,10] and retroviral particles have been detected in diseased animals [11]. The infection with the KoRV may be associated with chlamydial disease [12]. The virus infection is spreading from the north of Australia to the south, where uninfected animals still exist [4]. Whereas some years ago all koalas in Queensland but none of 26 animals tested on Kangaroo Island were found to infected [4], at present 15% of the animals on this island are already infected [13]. In addition, the ongoing epidemic is associated with an endogenization of the virus [3-5]. The unusually close phylogenetic relationship between KoRV and the pathogenic exogenous gibbon ape leukemia virus (GaLV) suggests that both viruses had a common progenitor and that the transmission was recent [14]. Meanwhile, it has become clear that KoRV entered the host population much earlier [15]. The rapid spread and the endogenization of KoRV in the koala population provides a unique opportunity to study the process of a retroviral invasion into a mammalian species and demands the immediate implementation of countermeasures. The diseases induced by the KoRV, such as lymphomas and immunodeficiency, account for up to 80% of deaths in captive animals [3] and the long-term effect of this epidemic on the entire koala population on top of the man-made fragmentation and restriction of the natural habitat is unpredictable. The fact that there are still uninfected animals offers the chance to protect a part of the koala population from the virus. One possibility would be to vaccinate KoRV-free koalas.

The envelope proteins of retroviruses are expressed on the surface of the virus particles and are the main targets for neutralizing antibodies. Since retroviruses integrate their genome as a DNA copy into the genome of the host cell where they may persist, neutralizing antibodies are, in contrast to the cellular immune responses, better suited to prevent infection and integration. We recently demonstrated the induction of neutralizing antibodies against the KoRV by immunization with the recombinant transmembrane envelope protein rp15E [16,17]. The antibodies recognized epitopes in the membrane proximal external region (MPER) as well as in the fusion peptide proximal region (FPPR). However, the titer of the neutralizing antibodies induced by the transmembrane envelope protein of KoRV was modest (1:10 to 1:20). Based on previous immunization studies using both the transmembrane and the surface envelope proteins of the porcine endogenous retrovirus (PERV) and the feline leukemia virus (FeLV), both closely related with the KoRV [18-25], we evaluated the potential of the surface envelope protein gp70 of KoRV to induce a neutralizing immune response. Furthermore, we combined gp70 with p15E in order to induce a broader repertoire of neutralizing antibodies. We demonstrate the induction of neutralizing antibodies by immunization with recombinant proteins and DNA corresponding to the sequence of both envelope proteins.

## Results

### Characterization of the antigens and DNA constructs

In order to induce neutralizing antibodies able to prevent infection with KoRV, rats were immunized with recombinant proteins corresponding to the transmembrane (p15E) and surface (gp70) envelope proteins of KoRV. Both proteins were produced in *E. coli*, rp15E in the form of the ectodomain with a molecular weight of 12 kDa, and rgp70/p52 as the entire surface envelope protein plus 25 amino acids of the N-terminal TM protein. Rgp70/p52 is nonglycosylated, has a molecular weight of 52 kDa and is analogous in sequence to the rgp70/p52 of the FeLV used in the commercial vaccine Leucogen [26] and to the recombinant rgp70/p52 of PERV and FeLV used in our previous experiments [19,20,24]. In addition, two goats were immunized with rp15E and one with rgp70/p52.

**Figure 1:**
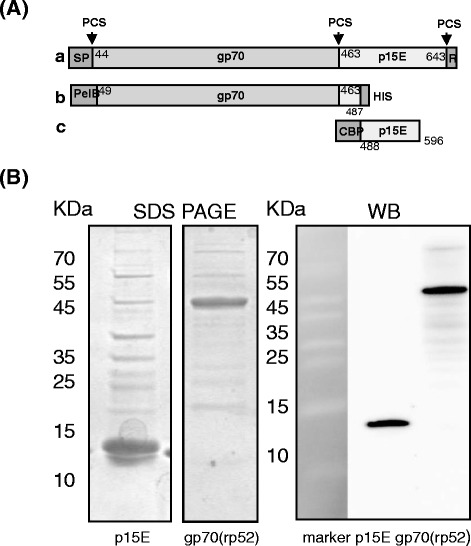
Characterization of the antigens used for immunization. **(A)** Schematic presentation of the viral envelope proteins. a, precursor envelope protein of KoRV (numbering according accession number AAZ99990.1), protease cleavage sites (PCS) are marked with arrow heads, SP-signal peptide. R-R peptide. b, recombinant gp70 as expressed using vector pET22b(+) with an N-terminal pelB leader sequence promoting translocation to the periplasma and a C-terminal His-tag. c, recombinant rp15E with N-terminal fused calmodulin binding protein (CBP) as expressed using the vector pCal-n, rp15E is the ectodomain of the transmembrane envelope protein, rgp70/rp52 is the entire surface envelope protein plus 25 amino acids from p15E. It is rp52 because it is not glycosylated. **(B)** SDS PAGE stained with Coomassie blue and Western blot (WB) analyses of the purified recombinant KoRV rp15E and rgp70/p52). KoRV proteins were detected using sera containing cross-reactive antibodies obtained by immunization of goats with recombinant rgp70/p52 and rp15E of PERV.

The proteins used for immunization reacted with the cross-reacting sera obtained by immunization with the corresponding recombinant proteins of PERV (Figure 1). Expression on the surface of transfected rat1 cells of gp70/gp85 encoded by the corresponding DNA construct was demonstrated by immunofluorescence and FACS analysis using goat sera against recombinant PERV rp15E and rgp70/p52 (not shown). To compare immunization strategies, we immunized with recombinant proteins and also directly inoculated DNA using the GeneGun. The immunization and bleeding schedule is given in the Methods.

### Analysis of binding antibodies by ELISA

Both strategies, e.g., immunizing with the recombinant proteins and with DNA, resulted in the induction of specific binding antibodies in all animals (Table 1). Immunization of rats and goats with the recombinant ectodomain of p15E (rp15E) induced binding antibodies with ELISA titers of 1.28x10^5^ - 2.56x10^5^ in rats and 5.12x10^5^ in goats. The titers of binding antibodies induced by immunization with recombinant rgp70/p52 were lower than those induced by immunization with rp15E. However, when an equimolar mixture of both recombinant proteins was used for immunization, a significantly lower titer of gp70 specific antibodies was observed, whereas the titers of p15E specific antibodies were similar to those after immunization with rp15E alone. Generally, DNA immunization induced titers of p15E-binding antibodies that were about ten times lower than those induced by recombinant protein whereas the titers of DNA-induced gp70-specific antibodies were comparable with, or even higher, than those seen using recombinant rgp70/p52 protein as immunogen (Table 1). Sera from animals infected with the empty pDisplay vector and a control serum obtained by immunization of a goat with recombinant rp27Gag of KoRV did not react in these ELISAs.

**Table 1:** **Titers of p15E and gp70 specific antibodies in sera obtained after immunization with recombinant rp15E, rgp70/p52 and DNA**

**Antigen/DNA**	**Antibodies anti-p15E**	**anti-gp70**
**Recombinant protein**		
gp70 (rat 1–4)	<500	128,000
p15E (rat 5–8)	128,000 - 256,000	<500
p15E + gp70 (rat 9–12)	64,000 – 1280,000	32,000 - 64,000
p15E (goat 31 and 46)	5120,000	<500
gp70 (goat 61)	<500	1280,000
p27Gag (goat 33)	<500	<500
**DNA**		
gp70 (rat 13–16)	<500	16,000 - 32,000
gp85 (rat 17–20)	4,000 - 8,000	8,000 - 16,000
pDisplay (rat 21–24)	<500	<500

### Analysis of neutralizing antibodies after immunization with recombinant proteins

**Figure 2:**
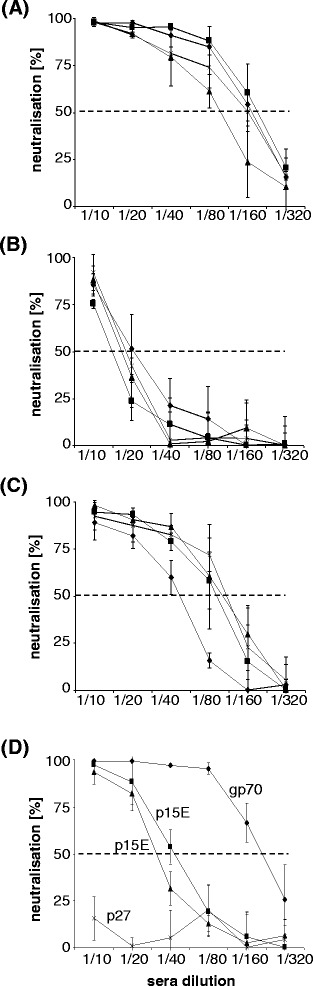
Neutralizing capacity of the induced antisera. Neutralization of KoRV by rat sera after immunization with recombinant rgp70/p52 protein **(A)**, recombinant zp15E protein **(B)** and both proteins together **(C)**. The titers of neutralizing antibodies in the sera from goats immunized with rgp70/p52, and rp15E are shown in **(D)**. A serum obtained by immunization with the recombinant rp27Gag (which is not neutralizing) was included for comparison. The dashed line indicates 50% neutralization.

**Figure 3:**
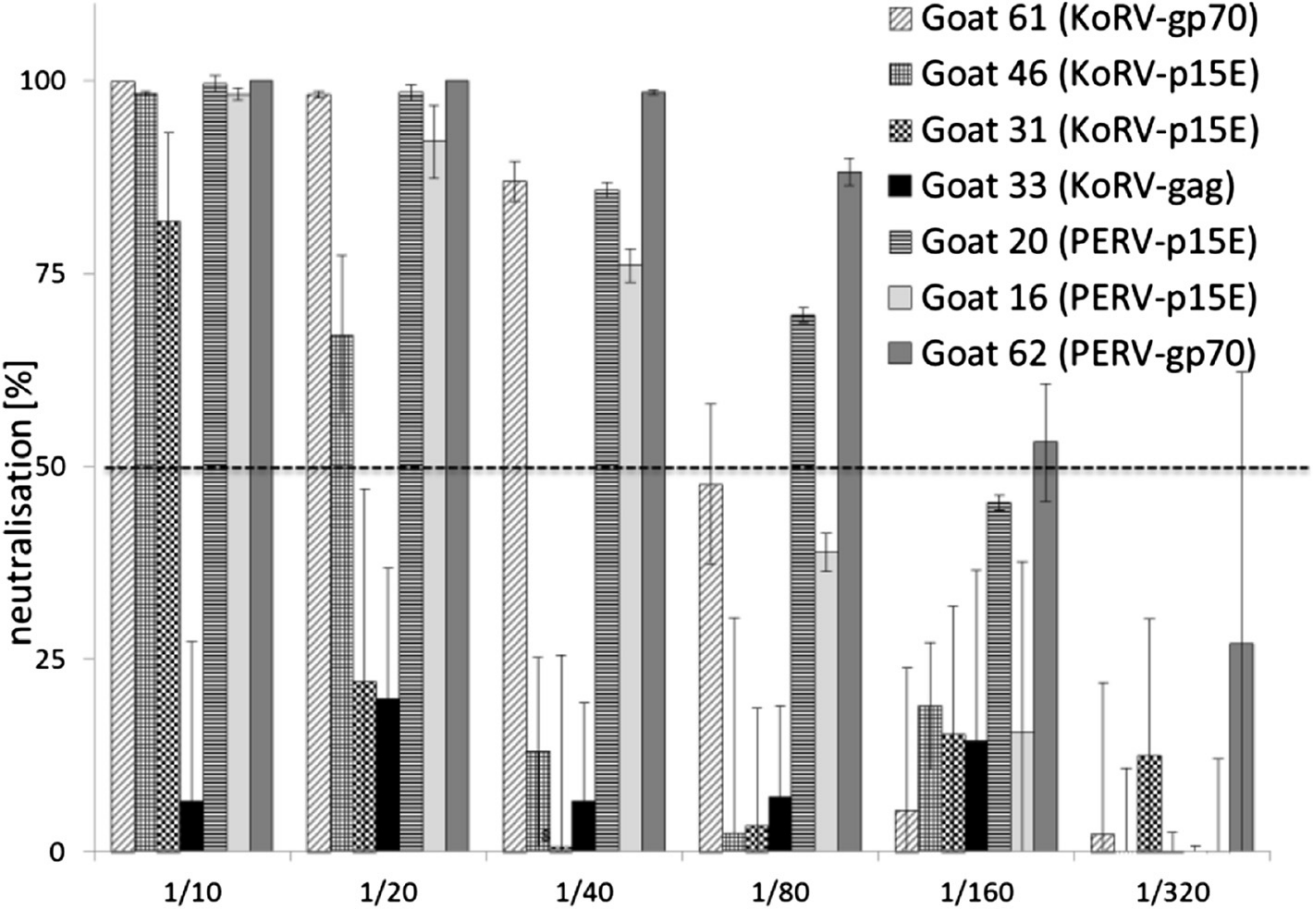
Cross-neutralizing activities. Neutralization of PERV with sera specific for p15E, gp70 and p27Gag of KoRV and PERV. The Y-axis indicates the percentage of reduction of integration of viral DNA into cellular DNA (infection) in the presence of immune sera. Sera were used at dilutions of 1:10 to 1:320 and integration was measured by real time PCR. The dashed line indicates 50% neutralization.

Immunization of rats with the recombinant transmembrane envelope protein rp15E, with the recombinant surface envelope protein rgp70/p52 and with a mixture of both resulted in the generation of neutralizing antibodies (Figure 2A,B,C). In comparison with the p15E-specific sera (1:20), the gp70-specific sera showed a significant higher titer of neutralizing antibodies (1:80 up to 1:160). The immunization with a mixture of both envelope proteins also resulted in neutralizing sera with titers in the same range (Figure 2C). The titers of the sera obtained after of immunization of goats were slightly higher (up to 1:320 for a serum against gp70 and 1:40 for one of two sera against p15E). A goat antiserum directed against p27Gag was not neutralizing (Figure 2D). Because KoRV and PERV are closely related, cross-neutralization was also investigated. Antisera specific for p15E of PERV and KoRV inhibited infection by the other virus, albeit to a lower extent (factor of 2–4, Figure 3). In the case of the p15E-specific sera this may be due to a sequence homology in the main epitope recognized by neutralizing antibodies in the MPER (Additional file 1: Figure S1). Using overlapping peptides epitope mappings were performed and for all neutralizing sera obtained by immunization with p15E of PERV [18,19], of FeLV [22-25] and of the KoRV [16] one epitope in the MPER of p15E was found which is highly conserved among all three viruses, FEG**WFN**. However, sera from goats immunized with the rp15E or rgp70/p52 of KoRV or PERV did not neutralize HIV-1. In addition, two HIV-1-specific broadly neutralizing monoclonal antibodies, 4E10 and 2 F5, directed against the transmembrane envelope protein gp41 of HIV-1 [27], did not neutralize KoRV or PERV (data not shown).

### Determination of neutralizing antibodies after DNA vaccination

**Figure 4:**
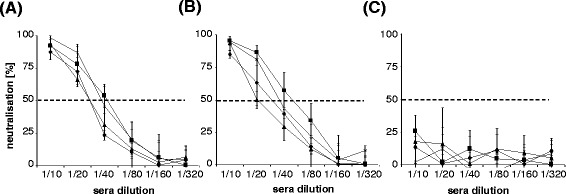
Neutralizing antibodies by DNA immunization. Neutralization of KoRV by rat sera obtained by GeneGun immunization with DNA coding for gp70 **(A)**, gp85 **(B)** and with the control empty vector **(C)**. The dashed line indicates 50% neutralization.

Immunizing with DNA coding for gp70 (Figure 4A) and for the corresponding to the precursor molecule gp85 (Figure 4B) resulted in neutralizing antibodies, whereas inoculation of the empty vector (Figure 4C) did not. The titers are lower compared to those seen after immunization with proteins.

## Discussion

Here we report for the first time the induction of neutralizing antibodies to KoRV using the recombinant envelope proteins rgp70/p52 and rp15E, either alone or in combination, and with DNA constructs coding for the surface envelope proteins gp70 and the precursor molecule gp85. Similar results were obtained in two species, i.e. rats and goats. Immunization with recombinant rgp70/p52 induced much higher titers of neutralizing antibodies compared to immunization with rp15E. A similar relationship was observed when immunizing with gp70 and p15E of PERV [19,20] and FeLV [24]. For immunization against gp70 a recombinant protein was used containing gp70 of the KoRV and a small N-terminal part of p15E. It therefore corresponds to the gp70 of FeLV which is used in the commercial vaccine Leucogen and induces solid protection from antigenemia and disease in immunized cats [26] and to the gp70 of PERV and FeLV used in our previous experiments [19,20,24]. Surprisingly, when we used a combination of rp15E and rgp70/p52, no increase in neutralization was observed. This is in contrast to similar immunization experiments with rp15E and rgp70/p52 of FeLV [24] and PERV [20]. However, in the case of FeLV and PERV, the difference between the neutralizing antibody titers induced by rgp70/p52 and by rgp70/p52 plus rp15E was only moderate. This may be due to the generally low levels of neutralizing activity induced by rp15E. There was, however, also a slight difference in the mode of application: whereas here rp15E and rgp70/p52 were applied as a mixture, in the experiments with PERV and FeLV the two proteins were applied separately at different sites [20,24].

The data obtained when immunizing with the envelope proteins of KoRV correlate well with the data obtained when immunizing with the envelope proteins of PERV [18-21] and FeLV [22-25]. The sera neutralized effectively in vitro and immunization of cats against FeLV was also effective in vivo, with 50% of the animals immunized with p15E and 100% of the animals immunized with gp70 or both antigens being protected from antigenemia [25]. To note, our antigen preparation containing rp15E and rgp70/p52 of FeLV as well as commercial vaccines against FeLV protect effectively cats from FeLV-related diseases [25,28,29]. However, none of the FeLV vaccines can induce sterilizing immunity and protect 100% of cats from infection [25,28,29]. To induce a more effective immune response by vaccination it may be necessary to change routes of vaccination and to use new adjuvants.

In all cases when immunizing with the recombinant transmembrane envelope protein rp15E of PERV, FeLV and KoRV [16-19,22-25], antibodies against the MPER and the FPPR were detected. At least in the case of PERV we demonstrated that only the antibodies directed against the MPER were neutralizing [21]. Interestingly, in the MPER of p15E of PERV, FeLV and KoRV an epitope was identified that has the same localization and a limited sequence homology with an epitope in the TM protein gp41 of HIV-1, recognized by the antibody 4E10 broadly neutralizing HIV-1 (N**WFN**IT, identical amino acids in bold) [27].

In a comparative study involving immunization with the TM protein of seven different retroviruses, including three gammaretroviruses (PERV, FeLV and KoRV), two lentiviruses (HIV-1 and HIV-2) as well as two foamyviruses (the feline FFV and the primate PFV), only the TM proteins of the gammaretroviruses induced neutralizing antibodies (for review see [30]). The reason for this remains unclear, although the fact that the TM proteins of the lentiviruses and foamyviruses are glycosylated and larger than those of gammaretroviruses such as KoRV may play a role. In addition, the foamy virus TM proteins contain more Cys amino acids that may be involved in the formation of several Cys-Cys loops. There are no vaccines currently available that can induce sterilizing immunity to retroviruses, e.g., all existing FeLV vaccines only prevent antigenemia and disease but not infection [25,28,29]. It may therefore be useful to induce cellular immune responses in addition to neutralizing antibodies or to further improve the induction of neutralizing antibodies.

Vaccination of uninfected koalas in Southern Australia or in international zoos may be the best way to stop the spread and ongoing endogenization of KoRV. If chlamydia infection of koalas is an opportunistic infection facilitated by KoRV-induced immunodeficiency, immunization against KoRV will also protect animals from chlamydia and other opportunistic infections. Here we demonstrated that two different vaccination strategies can be used as basis for a vaccination: (i) application of the recombinant envelope proteins p15E and gp70 and (ii) genetic immunization against p15E and gp70 via GeneGun. It would be interesting to compare these results with those obtained by DNA immunization with a protein boost. The development of a vaccine based on the recombinant envelope proteins p15E and gp70 will be a multistep process and the ability to induce neutralizing antibodies in koalas should first be analyzed. Additional immunization strategies possibly increasing the efficacy of the vaccine, e.g. prime boost immunization with DNA and proteins, should be developed. In a second step, protection *in vivo* could be evaluated. Although the main strategy here is to design a prophylactic vaccine in uninfected koalas, whether or not immunotherapeutic vaccination of animals already infected should also be addressed.

## Methods

### Recombinant rgp70/p52 of the KoRV

A sequence corresponding to the envelope protein of the KoRV (GenBank: AAZ99990.1) from amino acid 41 to 448 including the first 25 amino acids of the N-terminal part of the transmembrane envelope protein p15E was cloned. This protein exactly corresponds to the recombinant surface envelope protein of FeLV included in the commercial subunit vaccine against FeLV [31] and to the recombinant surface envelope protein of PERV and FeLV used in our experiments [19,20,25]. To obtain the clone, viral RNA was isolated from the supernatant of KoRV-infected 293 cells using the High Pure Viral RNA kit (Roche, Germany). Reverse transcription for cDNA synthesis was performed using the SuperScript One-Step-RT kit (Invitrogen) and the gp70 specific primers KoRV-For and KoRV-Rev (Table 2). The DNA was introduced into the pET22b(+) vector using the restriction sites EcoRI and SalI. The insert was verified by sequencing (GenBank: DQ174772.1). Comparison with another sequence (GeneBank: AF151794) revealed two amino acid exchanges, asparagine to histidine at position 408 and serine to proline at position 459, similar to a recently described replication-competent molecular clone KoRV522 [31]. *E. coli* BL21 DE3 cells were transformed and the expression of the protein p52, which is fused N-terminally to a 6xHis tag, was induced with 0.5 mM IPTG (24 h, 4°C). The 6xHis-tagged fusion protein was purified by Ni-NTA chromatography under denaturing conditions. The purity was verified by SDS-PAGE and Coomassie blue staining (Figure 1). For immunization, proteins were dialyzed extensively against phosphate buffered saline to remove guanidine hydrochloride.

**Table 2:** **Primers and probes used for PCR and real-time PCR analysis**

**Primer**	**Sequence**
KoRV-for	AGAATTCGAACCCTCACCAACCCATGACTC
KoRV-rev	AAAGTCGACGGCGGTCGAGCCGGTACC
KoRV-gp70-for	ATAAGATCTATGCTTCTCATCTCAAACCC
KoRV-gp70-rev	AAAGTCGACGGCGGTCGAGCCGGTACC
KoRV-gp85-rev	ATAGTCGACGGGGGAACGGTTGAACC
KoRV-5-for	CTAATAAAAGGGCCCATAGA
KoRV-6-rev	GTTGAACCATCCCTCGTACC
KoRV probe	6Fam-CCATGGATACAGACCTTAGGGCCC-BHQ1

### Cloning of gp70 and gp85 for DNA immunization

The sequences of KoRV-gp70 and -gp85 were cloned into the pDisplay vector (Invitrogen). For this, the sequences of gp70 or gp85 of KoRV were amplified from genomic koala DNA by PCR using the primers KoRV-gp70-forward, KoRV-gp70-reverse and KoRV-gp85-reverse (Table 2). To insert the gp70/gp85 sequences into the pDisplay vector, Bgl II (upstream) and Sal I (downstream) restriction sites were introduced. The gp70 constructs comprised the sequence of the entire surface envelope protein and the first 25 amino acids of p15E (aa1-448), similar to the recombinant protein, whereas the gp85 constructs contained gp70 and the entire ectodomain of the transmembrane envelope protein p15E (aa1-801). Expression of the antigens on the surface of transfected rat 1 cells was evaluated after 3 days by immunofluorescence and FACS analysis using antibodies against p15E and gp70.

### Immunization of rats and goats with recombinant rgp70/rp52, rp15E and rp27

Wistar rats and goats were obtained from the Federal Institute for Risk Assessment (Berlin, Germany). The animals were inoculated twice intramuscularly and subcutaneously (at weeks 0 and 3) with 0.25 mg of the purified recombinant rgp70/p52 (emulsified 1:1 in Freund’s adjuvant). Immune sera were obtained four weeks after the last immunization. Goats #31 and #46 were immunized with rp15E, goat #61 with rgp70/p52. For control, goat #33 was immunized with recombinant rp27Gag.

### DNA immunization of rats

Plasmids were purified using the Endo-Free-Maxi Prep Kit (Qiagen, Germany) and precipitated onto gold particles (Ø 0.9 μm, 2.5 μg DNA/1 mg gold). The coated particles were inoculated into the shaved abdominal skin of 8-week-old female Wistar rats using the Helios Gene Gun (Bio Rad, Munich, Germany) with a helium impulse at 300 psi. Each rat received a triple application (1 μg/shot). Boosts were performed on days 28 and 56 post primary immunisation. Immune sera were obtained 84 day after the first immunization.

### Western blot analyzes

Western blot analyses were performed as described [19]. The goat sera specific for p15E [16] and for gp70 of the KoRV obtained here were used at a dilution 1:1000, and secondary anti-goat antibodies (Dako) were used at 1:3000. In parallel goat sera specific for p15E, gp70, and Gag of PERV were used at a dilution 1:2500.

### ELISA

Titers of binding antibodies were determined by ELISA. To avoid false positive signals due to antibodies directed against *E. coli* proteins or sequences expressed from the pET22b(+) vector, sera were absorbed by overnight incubation with 200 μg/ml acetone precipitated proteins from an *E. coli* culture transfected with the empty vector at 4°C. Purified recombinant proteins (rp15E or rgp70/p52) diluted in water were added to microtiter plates (100 ng/well) (Nunc Immuno-Maxisorb) and dried overnight at 37°C. After blocking with 10 % FCS in PBS for 2 h at 37°C and washing with PBS with 0.05 % Tween 20 (PBS-Tween), 50 μl serum diluted in blocking buffer (5x10^2^ - 1,024x10^6^) were added and incubated for 2 h at 37°C. After washing three times with PBS-Tween, the peroxidase-conjugated secondary antibody was added and incubated for 60 min at 37°C. Finally the plates were washed five times with PBS-Tween and freshly prepared o-phenylenediamine/H_2_O_2_ solution was added. The reaction was terminated with Titrisol (Merck) and the optical density (492/620 nm) was measured.

### Neutralization assays

A novel neutralization assay was established based on quantification by real time PCR of proviral DNA in 293 T target cells after 65 h incubation (37°C, 5 % CO_2_) with virus supernatant and serum. For this, 100 μl of a cell suspension containing 1x10^5^/ml were plated into 96-well microtiter plates. One day later, cells were infected with 80 μl cell-free KoRV virus stock (1x10^2.66^ TCID_50_/ml) [16], preincubated for 15 min with 20 μl of heat-inactivated (60°C, 40 min) immune or preimmune serum in 2-fold dilutions (1:10 to 1:320). Quantitative real time PCR was performed using 3 μl proteinase K treated cell lysate [16] as template and self-designed specific primers KoRV-5-for, KoRV-6-rev with the KoRV probe (Table 2). The assays were performed in an MX3005 real time cycler (Agilent Technologies) (50 cycles, annealing at 56°C, 30 sec elongation phase). Significant neutralization was defined as a reduction in the amount of proviral DNA of over 50 %. The neutralizing activities of the sera analyzed were calculated using the formula: NT = 100-100/2^(ΔΔCt)^ [32]. All samples were measured in triplicate. To analyze whether cytotoxic components are present in the sera, a duplex real time PCR was established using the cellular gene GAPDH for co-amplification (Table 2, [33,34]). Sera which increased or decreased cell proliferation (+/− 0.5 Δct from the average of a pool of preimmune sera as control) were not included.

In addition, cross-neutralization of PERV with sera specific for p15E and gp70 of the closely related KoRV was analyzed. The neutralization assay for PERV was also based on a real-time PCR using virus repeatedly passaged on human 293 cells [35] and antisera against p15E (#16 and #20) and gp70 (#62) [18,19] as positive controls. The neutralization assay for HIV-1 IIIB was performed as described [33,34] using the broadly neutralizing antibodies 4E10 and 2 F5 as positive controls.

## Additional file

Additional file 1: Figure S1. Amino acid comparison of the MPER of different PERVs and KoRV. Conserved amino acids are in bold. The epitope recognized by antibodies neutralizing PERV is framed [18,21] and accession numbers are given.

## Abbreviations

FeLV: Feline leukemia virus
KoRV: Koala retrovirus
PERV: Porcine endogenous retrovirus
HIV: Human immunodeficiency virus
