# Supplementary material for: Induction of neutralizing antibodies specific for the envelope proteins of the koala retrovirus by immunization with recombinant proteins or with DNA
Source: Virol J. 2015 Apr 30;12:68. doi: 10.1186/s12985-015-0296-2 (PMC4429407; doi:10.1186/s12985-015-0296-2)
Supplement: Additional file 1: Figure S1. — Amino acid comparison of the MPER of different PERVs and KoRV. Conserved amino acids are in bold. The epitope recognized by antibodies neutralizing PERV is framed [18,21] and accession numbers are given. [file 12985_2015_296_MOESM1_ESM.pptx]

## Slide 1
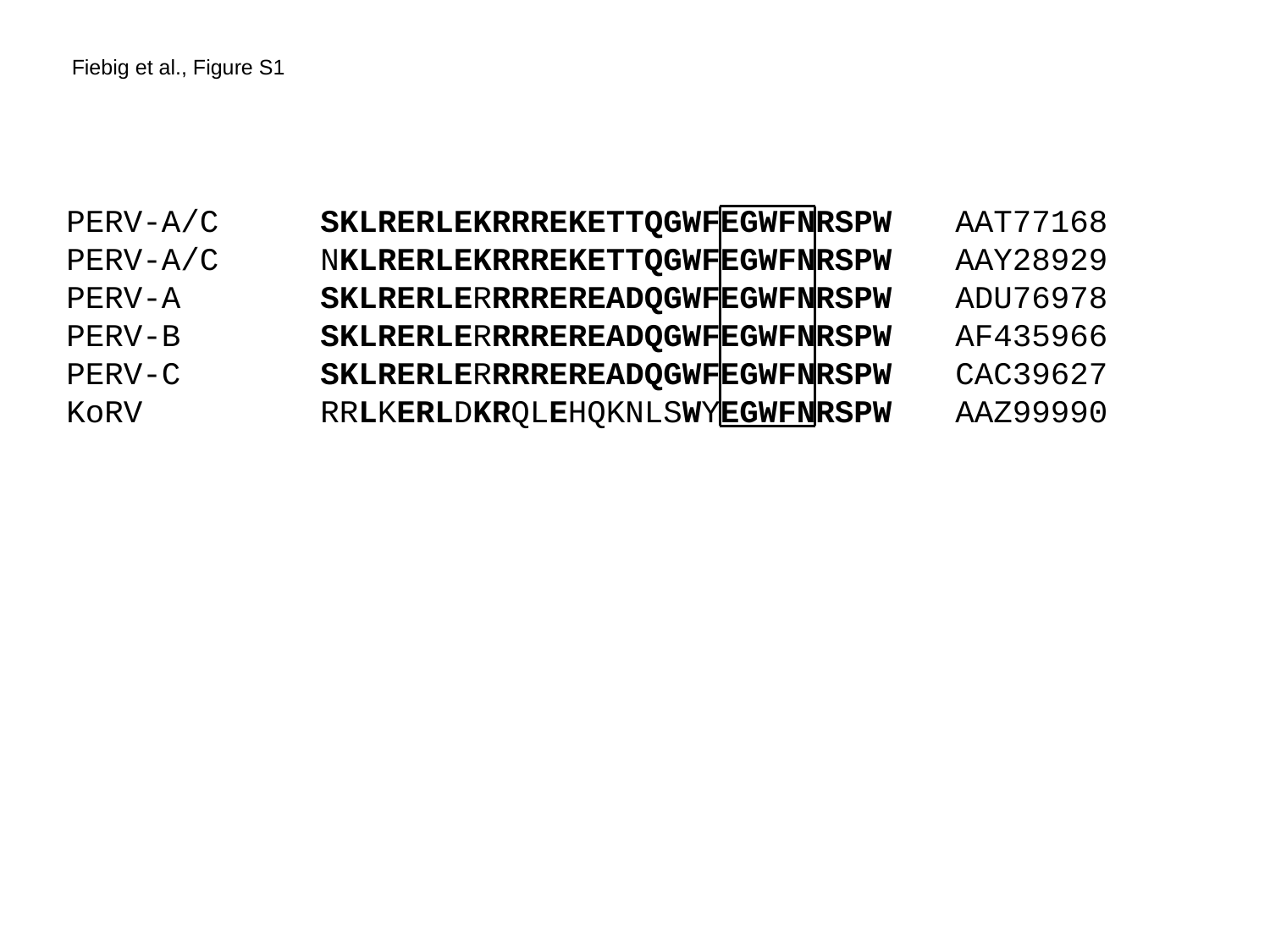

Fiebig et al., Figure S1
PERV-A/C 	sklrerlekrrrekettqgwfegwfnrspw 	AAT77168
PERV-A/C 	nklrerlekrrrekettqgwfegwfnrspw	AAY28929
PERV-A		sklrerlerrrrereadqgwfegwfnRSPW	ADU76978
PERV-B		sklrerlerrrrereadqgwfegwfnrspw	AF435966
PERV-C 	sklrerlerrrrereadqgwfegwfnrspw 	CAC39627
KoRV		rrlkerldkrqlehqknlswyegwfnrspW 	AAZ99990
